# Microfabricated Microbial Fuel Cell Arrays Reveal Electrochemically Active Microbes

**DOI:** 10.1371/journal.pone.0006570

**Published:** 2009-08-10

**Authors:** Huijie Hou, Lei Li, Younghak Cho, Paul de Figueiredo, Arum Han

**Affiliations:** 1 Department of Electrical and Computer Engineering, Texas A&M University, College Station, Texas, United States of America; 2 Department of Plant Pathology and Microbiology, Texas A&M University, College Station, Texas, United States of America; 3 School of Mechanical Design and Automation Engineering, Seoul National University of Technology, Seoul, Korea; 4 Department of Veterinary Pathobiology, Texas A&M University, College Station, Texas, United States of America; The Research Institute for Children at Children's Hospital New Orleans, United States of America

## Abstract

Microbial fuel cells (MFCs) are remarkable “green energy” devices that exploit microbes to generate electricity from organic compounds. MFC devices currently being used and studied do not generate sufficient power to support widespread and cost-effective applications. Hence, research has focused on strategies to enhance the power output of the MFC devices, including exploring more electrochemically active microbes to expand the few already known electricigen families. However, most of the MFC devices are not compatible with high throughput screening for finding microbes with higher electricity generation capabilities. Here, we describe the development of a microfabricated MFC array, a compact and user-friendly platform for the identification and characterization of electrochemically active microbes. The MFC array consists of 24 integrated anode and cathode chambers, which function as 24 independent miniature MFCs and support direct and parallel comparisons of microbial electrochemical activities. The electricity generation profiles of spatially distinct MFC chambers on the array loaded with *Shewanella oneidensis* MR-1 differed by less than 8%. A screen of environmental microbes using the array identified an isolate that was related to *Shewanella putrefaciens* IR-1 and *Shewanella* sp. MR-7, and displayed 2.3-fold higher power output than the *S. oneidensis* MR-1 reference strain. Therefore, the utility of the MFC array was demonstrated.

## Introduction

Microbial fuel cells (MFCs) are devices that generate electricity from organic compounds through microbial catabolism [1], [2], [3]. A typical MFC contains an anaerobic anode chamber and an aerobic cathode chamber separated by a proton exchange membrane (PEM), and an external circuit connects the anode and the cathode [4], [5]. Electrochemically active microbes (“electricigens”) reside within the anaerobic anode chamber. Electrons, generated during microbial oxidization of organic compounds, are delivered to the MFC anode via microbial membrane-associated components [3], [6], soluble electron shuttles [7], [8], or nanowires [9], [10]. Biofilms that support close physical interactions between microbial membranes and anode surfaces are also important for MFC power output [11]. Electrons flow from the anode to the cathode through the external electrical circuit. In parallel, protons generated at the anode diffuse through the PEM and join the electrons released to the catholyte (e.g. oxygen, ferricyanide) at the cathode chamber [1]. This electron transfer event completes the circuit.

MFCs have generated significant excitement in the bioenergy community because of their potential for powering diverse technologies, including wastewater treatment and bioremediation devices [12], [13], autonomous sensors for long-term operations in low accessibility regions [14], [15], mobile robot/sensor platforms [16], microscopic drug-delivery systems [17] and renewable energy systems [18]. In addition, MFCs hold significant promise for supporting civilian and combat operations in hostile environments [16]. Therefore, the development of efficient MFCs that are capable of producing high power densities remains an area of intense research interest. However, economical applications of existing MFCs are limited due to their low power output [19], which ranges from 100 to 1000 W/m^3^ [20], [21].

Important strategies for enhancing MFC performance include engineering optimized microbes (and microbial communities) for use in these devices [22] and improving cultivation practices for these organisms [23], [24]. To date, detailed description of individual microbe performance in MFCs has been limited to a surprisingly small number of organisms [25]. MFCs that are fed by sediment and wastewater nutrient sources and that exploit mixed microbial consortia for electricity generation have been described [26], [27]. However, with the conventional two-bottle MFCs, characterization of the electrochemical activities of the microbial species in these consortia has not been possible because these conventional MFCs are not suitable for parallel analyses due to their bulkiness. To address this issue, MFC systems that support parallel, low cost, and reproducible analysis of the electrochemical activities of diverse microbes are required. High throughput microarrays, including DNA microarrays, protein microarrays, and cell arrays, are powerful platforms for screening and analyzing diverse biological phenomena [28]. Various MFC platforms, including miniature MFC devices that enable parallel comparison of electricity generation in MFCs, are emerging [29], [30]. However, state of the art microfabrication and highly integrated parallel measurement approaches [31], [32] have not yet been exploited to construct an MFC array with highly consistent architecture and performance.

Here we describe our development of a compact and user-friendly MFC array prototype capable of examining and comparing the electricity generation ability of environmental microbes in parallel. The parallel analyses platform can greatly speed up research on electricigens. Importantly, the array was fabricated using advanced microfabrication approaches that can accommodate scale-up to massively parallel systems. The MFC array consisted of 24 integrated cathode and anode pairs as well as 24 cathode and anode chambers, which functioned as 24 independent miniature MFCs. We validated the utility of our MFC array by screening environmental microbes for isolates with enhanced electrochemical activities. Our highly compact MFC array enabled parallel analyses of power generation of various microbes with 380 times less reagents, and was 24-fold more efficient than conventional MFC configurations. This effort identified a *Shewanella* isolate that generates more than twice as much power as the reference strain when tested in both conventional and microfabricated array formats.

## Results and Discussion

**Figure 1:**
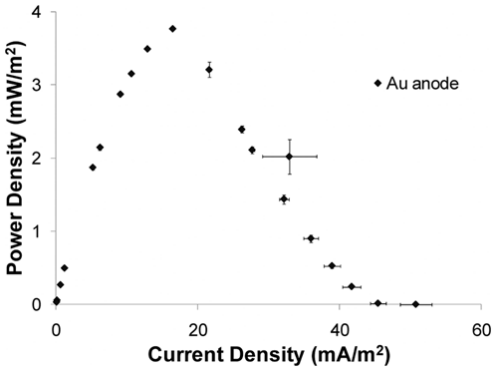
Au working as anode of MFC. Power density vs. current density from an MFC with gold anode (n = 3).

We recognized that selection of an appropriate anode material would be critical to the successful development of an MFC array and therefore initiated our studies by examining the performance of a commonly used anode material, carbon cloth in comparison to gold in a conventional MFC device (Fig. 1). We used the model facultative anaerobe *S. oneidensis* MR-1 for these experiments because this organism had previously proven useful for the development of MFC applications [37]. MFC power output was monitored for five hours after bacteria were introduced into the device. When the MFC was loaded with a 20 KΩ resistor, the gold electrode supported maximal power density of 3.77±0.02 mW/m^2^ at a current density of 16.47±0.04 mA/m^2^. Supporting Information (*SI*) Figure S1 shows power density as a function of current density with a carbon cloth anode. In this configuration, a high standard deviation was observed (10% deviation). However, the MFC with gold anode displayed greater reproducibility (3.1% deviation). The open circuit voltage (OCV) of MFCs containing gold and carbon cloth anodes was 514±12 mV (mean±SE, n = 3) and 538±51 mV (mean±SE, n = 4), respectively. These results indicated that the OCV of the MFC with the gold anode was comparable to the corresponding OCV with the carbon cloth anode. However, OCV measurements with the carbon cloth anode displayed greater variance.

An important hurdle to overcome in the development of MFC array systems is the identification of an electrode material that is durable, conductive, biocompatible, and easily fabricated [33]. Graphite, in the form of carbon cloth or graphite felt, has typically been the material of choice for the construction of MFC anodes, and conductive elements such as manganese, iron, quinines, and neutral red have been incorporated in graphite electrodes to significantly increase power output [33], [34]. However, graphite is not suitable for microfabricated MFC array systems [35]. The surfaces of graphite electrodes are non-uniform and difficult to pattern in small-scale devices. This non-uniformity thwarts efforts to compare performances between individual miniaturized MFCs. In addition, graphite materials are not compatible with most microfabrication technologies. Recently, gold has been identified as a potential material for MFC anode development [35]. Gold is highly conductive, can be vapor deposited, and is compatible with a wide array of conventional microfabrication modalities [36]. Thus, gold is a very attractive anode candidate for the development of an MFC screening platform. Our result showed that the MFC using gold as the anode material gave more reproducible results than its carbon cloth counterpart, a critical feature for side-by-side comparison in the MFC array. We therefore used gold as the anode material to develop the MFC array prototype.

Biofilms, when established on the anode of MFCs, enhance MFC performance when some microbial systems (including *S. oneidensis* MR-1) are employed. The enhanced performance has been suggested to result from the enhanced ability of biofilms to exploit close physical contacts between microbial membranes and the anode surface for electron transfer [11]. To investigate whether biofilms form on the surface of gold electrodes, light and fluorescence microscopy images (Figure S2) of the electrode were captured 1 hour and 5 hours post-inoculation (PI). One hour PI, microbes started attaching to the gold electrode surface (Figure S2*A*). Five hours later, an attached *S. oneidensis* biofilm was observed (Figure S2*B*). Scanning electron micrographs of the electrode surface confirmed microbial attachment (Figure S2*C,D*). Therefore, gold electrode supports *S. oneidensis* biofilm formation, and moreover, enables reproducible and consistent electrochemical activity to be measured when this model organism is used.

**Figure 2:**
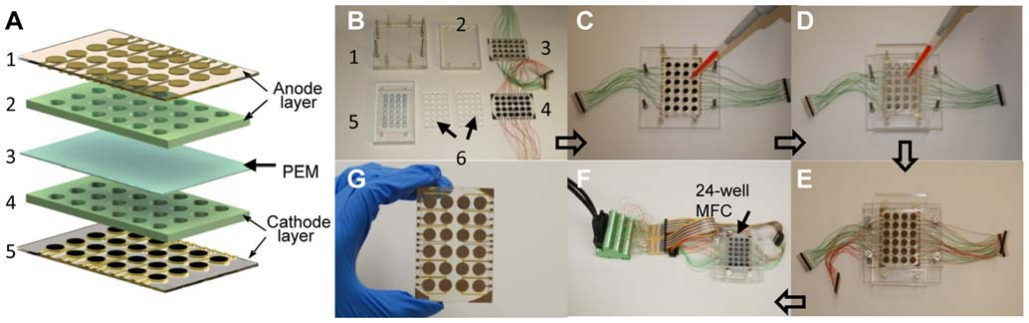
Illustration of the MFC array and its assembly steps. (A) Illustration of 24-well microbial fuel cell (MFC) array. Composed of an anode layer (1: anode electrode layer, 2: anode well layer), a proton exchange membrane (3: PEM), and a cathode layer (4: cathode well layer, 5: cathode electrode layer). (B)–(F) Microbial fuel cell array assembly. (B) Individual layers of the MFC array: acrylic support frames (1& 2), cathode layer (3), anode electrode substrate (4), anode well layer (5 sandwiched by 6). (C) Assembly of the acrylic frame (1) with the cathode layer (3), followed by cathode solution loading. (D) Anode well layer and PEM assembly followed by microbe loading. (E) A fully assembled MFC array with the anode electrode layer (4) and acrylic frame (2) capping the anode wells. (F) Fully assembled MFC array connected to load resistors and a data acquisition system. (G) An MFC array device with no acrylic frame.

**Figure 3:**
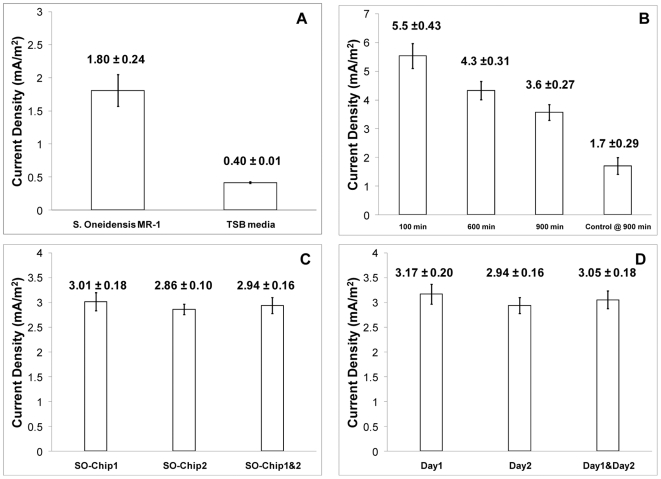
Characterization of current generated by *S. oneidensis* MR-1 in an MFC array. (A) Current densities generated by *S. oneidensis* MR-1 with PBS as the cathode solution at 350 min, TSB medium was used as the negative control (n = 4). (B) Repeatability of current densities generated by *S. oneidensis* MR-1 with ferricyanide as the cathode solution at different times after loading. TSB medium was used as the negative control (n = 5). (C) Chip-to-chip repeatability of current densities generated by *S. oneidensis* MR-1 with ferricyanide (100 mM) as the cathode solution at 1000 min (n = 4 for each chip). (D) Batch-to-batch repeatability of current densities generated by *S. oneidensis* MR-1 with ferricyanide (100 mM) as the cathode solution at 1000 min (n = 8 for each day). Means and standard errors were indicated above the bars (mean±SE).

We were encouraged by our finding that gold electrodes can be employed in MFC devices, and exploited this material to develop an MFC array (Fig. 2*A*). The array was successfully microfabricated using micromachining and soft lithography techniques (Figure S3). Performance and reproducibility of the MFC array were initially assessed by loading *S. oneidensis* MR-1 into the device and then measuring the electrical output. The current densities for negative control (un-inoculated medium) and *S. oneidensis* MR-1 chambers were 0.40±0.01 mA/m^2^ (mean±SE, n = 4) and 1.80±0.24 mA/m^2^ (mean±SE, n = 4), respectively (Fig. 3A). Therefore the MFC array reproducibly measured the electrochemical activities of this microbial system (less than 14% of variance).

To increase the current, we used 100 mM ferricyanide at the cathode as the electrolyte due to the higher concentration of the electron acceptor in the cathode solution [4]. Under this condition, *S. oneidensis* MR-1 produced a current density of 5.54±0.43 mA/m^2^ (mean±SE, n = 5) 100 minutes after loading microbes into the device (Fig. 3*B*). Over time, the current density gradually dropped, but remained higher than that of the negative control wells containing medium only. The electricity generation profiles of spatially distinct wells measured at multiple time points during 15 hours of operation differed by less than 8%, demonstrating that individual wells of the MFC array displayed comparable performance characteristics.

Performances of the same microbial culture were also compared in different MFC arrays. Two arrays with the same configuration were tested with the same microbial culture (OD_600_ = 0.8) and showed current density of 2.94±0.16 mA/m^2^ (mean±SE, n = 8) (Fig. 3*C*). Thus the MFC arrays showed chip-to-chip variances of less than 5.4%. Performances of the same chip with microbial cultures of the same concentration (OD_600_ = 0.8) prepared on different days were also examined (Fig. 3*D*). The current densities on 2 different days were 3.05±0.18 mA/m^2^ (mean±SE, n = 16), showing a 5.9% variance. Therefore, the MFC array provided a platform for reproducible experimentation.

**Figure 4:**
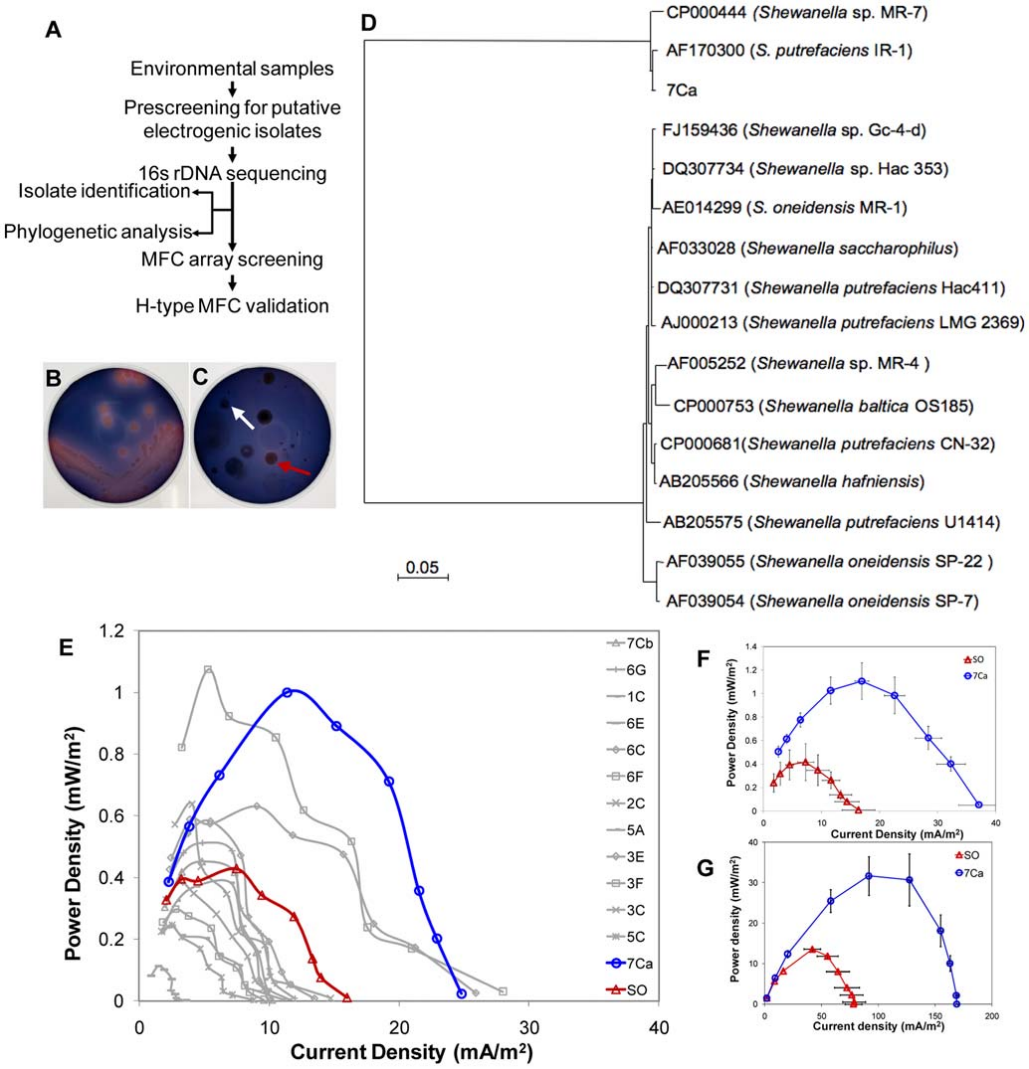
Environmental sample screening with the MFC array. (A) A schematic representation of the screening process for the environmental microbes with enhanced electricity generation capacities. (B)&(C) Electrochemically active microbes cause discoloration of an azo dye, reactive black 5 in the nutrient agar screening plate. (B) *S. oneidensis* MR-1 was used as the control; (C) A representative plate with a putative microbe isolate indicated by the red arrow and a non-putative microbe isolate indicated by the white arrow. (D) Phylogenetic tree based on 16S rDNA sequences indicating the relationship of various *Shewanella* species. (E)–(G) Screening of environmental isolates using the 24-well MFC array. (E) Screening of 13 environmental isolates with *S. oneidensis* MR-1 as the positive control (SO, red) using two 24-well MFC arrays in parallel. The average power density of two replicates was shown for each isolate. (F) The power density of 7Ca compared to the *S. oneidensis* MR-1 reference strain (n = 8). (G) Validation of current generation by 7Ca and *S. oneidensis* MR-1 in conventional MFCs (n = 4).

Encouraged by the performance and reproducibility of the MFC array, we examined whether the device could be employed to quickly screen environmental microbes for individual isolates that display enhanced electrochemical activities. A schematic representation of the screening process is shown in Fig. 4*A*. We pre-screened ∼12,000 microbes derived from environmental (water and soil) samples on solid medium containing Reaction Black 5, an azo dye that indicates electrochemically active organisms (Fig. 4*B,C*) [39]. 16S rDNA sequencing analysis of 26 hits obtained from the pre-screening plates revealed that the majority of the isolates (n = 10) were members of the *Bacilli* and γ-proteobacteria classes (Table 1 and Figure S4). We then exploited the MFC arrays to characterize the electrochemical activities of several isolates. One isolate 7Ca reproducibly showed 266% higher power output than the *S. oneidensis* MR-1 reference strain in both the primary screening (Fig. 4*E*) and the secondary confirmation with more replicates in the MFC arrays (Fig. 4*F*) (polarization curve shown in Figure S5). Phylogenetic analysis demonstrated that 7Ca was most closely related to *Shewanella putrefaciens* IR-1 (98% sequence similarity) and *Shewanella* sp. MR-7 (98% sequence similarity) (Fig. 4*D*). The high power generation capability of 7Ca was further validated in 24-hour trials in a conventional H-type MFC system (Fig. 4*G*) [6]. In our specific conventional MFC configuration, the maximum current density of 7Ca was 169.00±10.60 mA/m^2^, which was 217% higher than the current density (78.00±7.30 mA/m^2^) generated by the *S. oneidensis* MR-1 control. The maximum power density of 7Ca was also 233% higher than this reference strain. Although we used gold as the anode material in the MFC array and carbon cloth as the anode material in the H-type MFC system, the power density increases of 266% in the MFC array and 233% increase in the H-type MFC system showed that findings in our MFC array system can be translated to larger scale conventional systems. Thus, insights garnered using gold anodes in miniaturized MFCs can be transferred to conventional MFC formats using carbon anodes.

**Table 1:** Identities of environmental isolates obtained from pre-screening.

Isolate	Top Hit (Genbank Number)	Identity%
1B	*Bacillus* sp. RC33 (FJ263036.1)	100
1C	*Bacillus* sp. W1-17 (FJ560473.1)	100
1D	*Bacillus niacini* strain YM1C7 (EU221338.1)	97.93
2A#	*Enterobacter* sp. CTSP29 (EU855207.1)	99
2B	*Bacillus* sp. SX51 (DQ227355.1)	
2C, 3B, 4A, 5A, 5B	*Bacillus thuringiensis* serovar tenebrionis (EU429671.1)	100
3A	Bacterium 3A13 (DQ298760.1)	99
3C	*Arthrobacter* sp. FB24 (EU147009.1)	100
3E, 3F	*Bacillus* sp. A1 (2008) (FJ535468.1)	100
5C	*Bacillus* sp. BM1-4 (FJ528077.1)	100
6A, 6C*	*Pseudomonas putida* strain J312 (EF203210.1)	98.50
6B* ^,^ #	*Stenotrophomonas maltophilia* strain CMG3098 (EU048328.1)	98.99
6E*	*Pseudomonas plecoglossicida* strain S19 (DQ095907.1)	98.52
6F*	*Pseudomonas* sp. lm10 (EU240462.1)	98.72
6G*	*Pseudomonas* sp. GNE25 (AM397659.1)	95
7A* ^,^ #	*Aeromonas* sp. LD151 (AM913921.1)	97.34
7Ca*	*Shewanella* sp. Hac353 (DQ307734.1)	99
7Cb	*Bacillus pumilus* strain TPR18 (EU373436.1)	99
8A* ^,^ #	*Paenibacillus* sp. oral clone CA007 (AF385540.1)	92
8B* ^,^ #	*Aeromonas hydrophila* strain IB343 (EU770277.1)	99

* isolates belonging to γ-proteobacteria (10 out of 26 isolates).

# putative pathogenic isolates not subject to electricity generation screening using MFC array.

We have demonstrated that a microfabricated MFC array system can be exploited to rapidly screen and characterize microbial electrochemical activity. The universal design of our system has several attractive features. First, the microbe culture chamber was easy to assemble and reusable. The PDMS and electrode could be used at least 10 times without degradation and the acryl anode chamber could be used more than 10 times with proper cleaning. Therefore, the device has the potential for widespread adoption. Second, because the individual MFC chambers in the array hold a small volume, 380-fold fewer reagents are required than conventional MFC devices. Third, the universal design of the array makes it possible to easily change the anode and cathode to quickly explore new electrode materials to further optimize the MFC architecture. Fourth, the device can support factorial experiments in which several variables are tested and compared simultaneously. This feature will dramatically accelerate the pace of electricigen research. Fifth, the highly scalable approaches used to microfabricate the array set the stage for the development of next-generation parallel devices with more than 1,000 wells. Finally, the array provides a platform for MFC performance to be assessed in parallel (increasing MFC experimental throughput by 24-fold). We exploited this feature to identify and characterize electricigens with high electrochemical activities. In this regard, the fact that several microbes with enhanced electrochemical activity were rapidly uncovered in our screen indicates that the natural environment constitutes a plentiful reservoir for mining electricigens. For example, our screen uncovered four *Pseudomonas* sp. (Table 1 and Figure S4) that produce phenazine-based metabolites that can serve as electron shuttles [40], [41]. Moreover, the screen resolved *Bacillus* sp. and *Aeromonas hydrophila* that have been reported to be present in microbial consortia of MFCs [26]. A *Shewanella* isolate 7Ca, which generated power density that was 2.3-fold above the reference strain, was also uncovered (Fig. 4*E*).

It is intriguing to speculate on the molecular mechanisms that contribute to the enhanced electrochemical activity of 7Ca. For example, the presence of gain of function mutations affecting biofilm formation, nanowire formation, cell membrane electron transfer, metabolic and respiration capacities, regulatory components for these functions, and/or combinations of all the above, could contribute to the observed power output. In this regard, it is notable that previous genetic studies in *S. oneidensis* MR-1 revealed several genes that are directly involved in electricity generation, including *mtrA, mtrB, omcA/mtrC, cymA, fur*, and *crp*. Deletion of these genes caused severe reductions in current production [42]. Gain of function mutations at these candidate loci may therefore confer enhanced MFC power generating properties. Similarly, an engineered strain of *Geobacter sulfurreducens* generated by Izallalen et al. displayed enhanced electrochemical activity due to increased respiration rates [22]. Despite these molecular insights, there has been a paucity of functional studies that directly measure the electrochemical activities of environmental microbes. In fact, the electrochemical activities of only a handful of microbial species have been characterized in MFC systems [3]. Although MFCs using wastewater treatment and sediment nutrient sources have defined electrochemically active microbial consortia [43], the electrochemical activities of individual species within these consortia remain largely unexplored. It is likely that the MFC array system reported here will facilitate and accelerate these kinds of studies.

### Conclusions

A microfabricated MFC array, a compact and user-friendly platform for the identification and characterization of electrochemically active microbes, was developed. The MFC array consisted of 24 integrated anode and cathode chambers, which functioned as 24 independent miniature MFCs. The electricity generation profiles of spatially distinct MFC chambers on the array loaded with *Shewanella oneidensis* MR-1 differed by less than 8%. The utility of the MFC array was demonstrated by screening environmental microbes and resulted in the identification of a microbe that displayed 2.3-fold higher power output than the *S. oneidensis* MR-1 reference strain. The MFC array consumed 380 fold less samples and reagents compared to a single H-type MFC, and 24 parallel analyses could be conducted simultaneously. We expect to further scale up the MFC array into a 96-well MFC array.

## Materials and Methods

### Twenty-four well MFC array design

Fig. 2*A* shows the schematic illustration of the MFC array. The array was microfabricated using micromachining and soft lithography techniques (Figure S3). The 24-well device was composed of layered functional compartments in which microbe culture wells were embedded. Each microliter-scale microbe culture well was combined with individually addressable anode and cathode electrodes and functioned as a separate MFC. The layers included anode electrodes, anaerobic microbe culture chambers (anode wells), a proton exchange membrane (PEM), cathode chambers, and cathode electrodes (Fig. 2*A*). The assembled device (Fig. 2*B–E*) with acrylic supporting frames was coupled to a load resistor circuit board and a digital multimeter through a computer controlled switch box module and a data acquisition system (Fig. 2*F*).

### Twenty-four well MFC array electrode layer microfabrication

An acrylic master mold having 4×6 arrays of pillars (diameter: 7 mm, height: 4 mm) was fabricated with a rapid prototyping machine (MDX-40, Roland Inc., Los Angeles, CA). PDMS precursor solution (Sylgard 184, Dow Corning, Auburn, MI) prepared by mixing base and curing agent at 10∶1 ratio (v/v) was poured onto the acrylic master mold. After curing for 30 min at 85°C, the resulting polymerized PDMS slab was peeled off, creating an inverse replica of the acrylic master mold. The cathode layer was prepared by aligning and permanently bonding a PDMS well layer onto a patterned electrode layer. Platinum loaded carbon cloth (10%, A1STD ECC, BASF Fuel Cell, Inc., Somerset, NJ) was cut to the size of a well (diameter: 7 mm) and bonded on top of the Au electrode pads in the cathode electrode layer using silver paste (Structure probe, Inc., West Chester, PA). Cathode and anode electrode layers of the 24-well MFC array were fabricated using standard microfabrication techniques (Figure S3*A* and *SI* for detailed fabrication steps). The fabricated cathode and anode electrode substrates had 24 individually addressable electrodes, each having an 8 mm diameter disk pattern. Wires were then soldered to all contact pads to provide electrical interconnects between the MFC arrays and the voltage measurement setup.

### Assembly of the MFC array system

The 24-well MFC array system consisted of a 24-cathode array layer, a cathode well layer, a proton exchange membrane, an anode well layer, and a 24-anode array layer. Cathode layer consisted of a PDMS well layer permanently bonded on an electrode layer. Pt loaded carbon cloth was cut to the size of the well (diameter: 7 mm) and bonded on top of the Au electrode pads. The anode layer consisted of three layers: two PDMS layers fabricated as described above and an acrylic layer (8 mm thick) having 4×6 arrays of through-holes (7 mm diameter) fabricated by a rapid prototyping machine. The two PDMS layers were placed on both sides of the acrylic layer. The rigid acrylic layer served as a support layer that could be clamped tightly in the subsequent assembly step. The cathode layer, activated proton exchange membrane, and the anode layer were then assembled together. To assemble these layers together, a top and bottom acrylic support frame that could be used to tightly clamp all layers together in between was cut out using a rapid prototyping machine. The sequence of images (Fig. 2*B–F*) shows how all parts of a 24-well MFC array system was assembled. See Methods S1 for detailed assembly steps.

### Organisms, media and growth conditions

*Shewanella oneidensis* MR-1 was obtained from American Type Culture Collection (ATCC, Manassas, VA). Environmental bacteria used for screening were isolated from eight different samples (soil and water) obtained from Lake Somerville (N30°30′09″ and E96°64′28″), Brazos River (N30°55′84″ and E96°42′24″; N30°62′64″ and E96°55′13″) and Lake Finheather (N30°64′93″ and E96°37′54″) around College Station, Texas. See Methods S1 for detailed culture protocols.

### Isolation and pre-screening of environmental microbes

We performed a pre-screening for electrochemically active microbes. Each diluted sample was plated on nutrient agar and incubated under anaerobic conditions. The resulting 50–100 microbial colonies per plate were then used for plating on nutrient agar containing 100 µM Reaction Black 5, an azo dye that resulted in dark blue color of the media. After 3 days of incubation, a total of 26 colonies formed discoloration halos out of about 1500 colonies plated for each of the eight environmental samples. The discoloration of the dye indicated reduction capability of the microbes. A total of 13 isolates were selected for MFC array screening. See Methods S1 for details. Un-inoculated medium was used as the negative control.

### 16s rDNA amplification and phylogenetic analyses for environmental isolates

Colony PCRs were performed using different environmental isolates as the templates. The PCR products were then purified and sequenced with primers 11F and 1492R. The 16S rDNA sequences were BLAST searched against the GenBank database and the top hit for each isolate were used for alignment and phylogenetic tree generation. Sequences of the 16S rDNA of 15 members of genus *Shewanella* similar to 7Ca were aligned and phylogenetic tree was constructed among selected *Shewanella*. A matrix of pairwise genetic distances by the maximum-parsimony algorithm and the neighbor-joining method was used to generate phylogenetic trees. See Methods S1 for details.

### MFC array characterization and data acquisition

Two characterization methods were used to evaluate electricity generation from each of the 24 MFC wells. First, 24 1 MΩ fixed load resistors were connected to each of the MFC wells and voltage across these resistors were recorded. Load resistance of 1 MΩ was selected for MFC characterization because power output of *S. oneidensis* MR-1 at this resistance was close to maximum and the fabricated MFC array showed a steady state current output much faster than using resistors with lower resistances. A switch box module having an integrated digital multimeter (PXI-2575, PXI-4065, National Instruments, Austin, TX) and controlled through LabView™ (National Instruments, Austin, TX) was used to continuously measure voltages across the 24 load resistors that were connected individually to the 24 MFC array. The measured voltages were converted to current densities (mA/m^2^, electrode area: 0.385 cm^2^) using Ohm's law, and power densities were calculated using P = VI/A (V: voltage, I: current, A: electrode area).

Full characterization of an MFC requires a current density versus power density plot, which can be obtained when measuring voltages across varying resistors. In this second characterization method, twenty-four 100 KΩ variable resistors (652-3296Y-1-104LF, Mouser Electronics, Mansfield, TX) were connected in series with twenty-four 2 MΩ variable resistors (652-3296Y-1-205LF, Mouser Electronics, Mansfield, TX) in pairs on a circuit board, connected correspondingly to the 24 MFC wells. For environmental screening of microbes using the MFC array, both the primary screening and the secondary confirmation started 1000 min after loading microbes into the MFC array loaded with 1 MΩ resistors. One, 10, 20, 50, 100, 200, 500, 1,000, and 2,000 KΩ loading resistors were used and voltages across these resistors were continuously recorded.

### Conventional H-type MFC setup and characterization and Microcopy

See Methods S1.

## Supporting Information

Methods S1 (0.04 MB DOC)

Figure S1 Power density vs. current density from an MFC with carbon cloth anode (n = 3).
(0.39 MB TIF)

Figure S2 Microscopy images of Au electrode. (A) After 1 hour of usage (light microscope). (B) After 5 hours of usage (fluorescent microscopy, DAPI staining). Microbes attached to the gold electrode could be clearly observed. (C) & (D): Scanning electron micrographs of microbes attached to the surface of the gold electrode after 5 hours in an MFC.
(4.63 MB TIF)

Figure S3 Fabrication steps of the MFC array. (A) Electrode layer (both cathode and anode) fabrication steps. 1. Titanium deposition; 2. Gold deposition; 3. Photoresist (PR) spin coating; 4. UV exposure of PR through a lithography mask; 5. PR developing; 6. Au and Ti etching; 7. PR removing. (B) PDMS layer fabrication steps via softlithography for cathode and anode well layers. 1. Acrylic master mold fabrication using a rapid prototyping tool; 2. PDMS mixing and pouring onto the acrylic master mold; 3. PDMS curing and peeling off.
(0.45 MB TIF)

Figure S4 Phylogenetic tree based on 16S rDNA sequences showing relationship within of the environmental isolates obtained in the pre-screening. Most environmental isolates were members of classes Bacilli or γ-proteobacteria.
(2.65 MB TIF)

Figure S5 Polarization curves of 7Ca (blue) and S. oneidensis MR-1 (SO, red) in the MFC array.
(0.12 MB TIF)
