# Supplementary material for: Microfabricated Microbial Fuel Cell Arrays Reveal Electrochemically Active Microbes
Source: PLoS One. 2009 Aug 10;4(8):e6570. doi: 10.1371/journal.pone.0006570 (PMC2718701; doi:10.1371/journal.pone.0006570)
Supplement: Methods S1 — (0.04 MB DOC) [file pone.0006570.s001.doc]

**Supplementary Materials and Methods**

**Cathode and anode electrode fabrication**

Glass substrates (50 mm × 75 mm) were cleaned in “piranha” solution (H_2_O_2_ : H_2_SO_4_ = 1 : 3 v/v) for 30 min, followed by thoroughly washing with de-ionized (DI) water and blow drying with N_2_. Titanium (thickness: 200 Å, deposition rate: 2 Å/sec) and gold (thickness: 1200 Å, deposition rate: 3 Å/sec) were deposited on cleaned glass slides using electron beam evaporation. Photoresist (Shipley S-1818, Rohm and Haas, Inc., Philadelphia, PA) was spin-coated on metal coated glass slides at 4000 rpm, baked at 100 °C oven for 10 min, then exposed to UV light through a photolithography mask for 18 sec using a mask aligner (MA6, SUSS Microtech, Germany) at an intensity of 8.5 mW/cm^2^ (wavelength = 320 nm), and then developed in a photoresist developer (MF^TM^ 319, Rohm and Haas, Inc., Philadelphia, PA) for 20 seconds. The exposed part of the Au and Ti layers were then removed in Au etchant (Type TFA, Transene Company Inc., Danvers, MA) and Ti etchant (HF : H_2_O = 1 : 300 v/v). Finally, the photoresist was removed with acetone and cleaned with isopropanal and DI water.

**Proton exchange membrane (PEM) treatment**

The PEM (Nafion 117^TM^, Ion-Power, Inc., New Castle, DE) was pretreated by immersion in 5 % H_2_O_2_ for 1 hour at 80 °C, rinsed and boiled in DI water for 1 hour at 80 °C, and then boiled in 0.5 M H_2_SO_4_ for 1 hour at 80 °C. After rinsing with DI water followed by another 1 hour boiling in DI water at 80 °C, the membrane was stored in DI water until use.

**Organisms, media, and growth conditions**

All bacterial cells were stored at -80 °C in tryptic soy broth (TSB, pancreatic digest of casein 1.7 %, enzymatic digest of soybean meal 0.3 %, glucose 0.25 %, NaCl 0.5 %, K_2_HPO_4_ 0.25 %, pH 7.3) supplemented with 15 % glycerol. Cells were streaked onto a TSB agar plate from storage. One of the resultant single colonies was inoculated into 5 ml of TSB liquid medium, and then shaken at 150 rpm for 48 h at 30 ˚C. To perform MFC array screening, the optical densities (OD_600_) for *S. oneidensis* and the environmental isolates were measured and adjusted to 0.8 before loading into the anode wells of the MFC array, and 100 mM ferricyanide in phosphate buffered saline (PBS, 0.8 % NaCl, 0.02 % KCl, 0.144 % Na_2_HPO_4_, 0.024 % KH_2_PO_4_, pH 7.4) was used as the cathode solution. For confirmation using the conventional MFC, 5 ml of the actively growing overnight cultures (OD_600_ = 0.7) were inoculated into 500 ml of TSB and incubated for 48 h (OD_600_ = 1.5) at 30 ˚C and then loaded into the anode chamber, and 100 mM ferricyanide in 100 mM sodium phosphate buffer (pH 7.0) was used as the cathode solution.

**Isolation and pre-screening of environmental microbes**

We performed a pre-screening for electrochemically active microbes. Each environmental soil sample was weighted in a 50 mL tube. To make sample suspension, 50 mL of sterile double distilled water were added in each tube and the tube was vortexed for 1 min. Microbial richness in each sample was determined by serial dilutions. Water samples were used for dilution directly. Specifically, 100 μl of each dilution was plated on nutrient agar with 100 μg/mL cycloheximide to eliminate fungal contaminations, and the plates were incubated for 3 days at 30°C under anaerobic conditions. The diluted samples that resulted in 50-100 microbial colonies per plate were then used for plating on nutrient agar containing 100 μM Reaction Black 5 (Sigma-Aldrich, St. Louis, MO), an azo dye that resulted in dark blue color of the media. After 3 days of incubation, a total of 26 colonies formed discoloration halos out of about 1500 colonies plated for each of the eight environmental samples. The discoloration of the dye indicated reduction capability of the microbes. A total of 13 isolates were selected for MFC array screening.

**16s rDNA amplification and phylogenetic analyses for environmental isolates**

Colony PCRs were performed using different environmental isolates as the templates. To amplify the 16S rDNA, primers 11F (GTTTGATCCTGGCTCAG) and 1492R (TACCTTGTTACGACTT) [1] were used and the thermal cycling program was as follows: 94 °C for 5 min, 35 cycles of 1 min at 94 °C, 45 sec at 48 °C and 2 min at 72 °C, followed by a final extension for 10 min at 72 °C. The PCR products were then purified with the QIAquick PCR Purification Kit (Qiagen, Valencia, CA) and sequenced with primers 11F and 1492R.

The16S rDNA sequences amplified from the environment isolates was BLAST searched against the GenBank database. The 16S rDNA sequences of the top hit for each isolate were used for alignment and phylogenetic tree generation for all the environmental isolates. Sequences of the 16S rDNA of 15 members of genus *Shewanella* similar to 7Ca were aligned and phylogenetic tree was constructed among selected *Shewanella*. A matrix of pairwise genetic distances by the maximum-parsimony algorithm and the neighbor-joining method was used to generate phylogenetic trees using the ClustalX software (version 2.0) [2].

**Conventional H-type MFC setup and characterization**

The conventional MFC setup comprised of two sterile glass containers (250 ml) with rubber gasketed glass bridges. The PEM was clamped between the two glass bridges. Each container had a cap with a 1 mm diameter opening through which wires were fed to make electrical contact with the anode and cathode. The anode opening was then sealed with a tape during experiments. The cathode cap remained open to allow oxygen exchange.

Gold substrate and carbon cloth were used as the anode materials. Gold was deposited on a clean 50 mm by 75 mm glass slide (Corning, NY) using an electron-beam evaporator to a thickness of 80 nm (deposition rate 5 Å/sec). The gold-coated glass slide was then cut to 2.5 cm by 3 cm size substrates (surface area: 7.5 cm^2^), followed by rinsing with acetone, isopropyl alcohol, and deionized (DI) water.

For experiments using carbon electrodes, carbon cloth without waterproofing (B1A, BASF Fuel Cell, Inc., NJ) and carbon cloth loaded with platinum (A1STD, 0.5 mg/cm^2^ loading with 10 % Pt, BASF Fuel Cell, Inc., NJ) were used as the anode and cathode materials, respectively. Carbon cloth without waterproofing and platinum loaded carbon cloth were used as the anode and cathode materials, respectively. The carbon electrode size for both the anode and cathode was 2.5 × 3 cm (surface area: 7.5 cm^2^). Wires attaching the electrodes were connected to a multimeter and a resistor board. All electrodes were sterilized in 70 % ethanol for 10 min before use.

Open circuit voltage (OCV) of the MFC was measured using a potentiostat with data acquisition capability (EC Epsilon, BASi, Inc., IN).

To characterize the power output, the MFC was connected to a resistance selector (Ohm-Ranger, Ohmite Mfg. Co., IL). For power curve generation, 0, 0.1, 0.5, 1, 2.5, 5, 10, 40, 100 and 500 KΩ loading resistors were used and current recorded with a digital multimeter (34410A, Agilent Technologies, Inc., CA and 8840A, Fluke, WA) connected to a data acquisition setup (GPIB computer interface and LabView^TM^ software, National Instruments, Inc., TX). Current was recorded for 16 h without load resistor first to stabilize the MFC, and then recorded continuously with each resistor applied for at least 20 min. Power was calculated using Ohm’s Law (P = I^2^R).

**Microscopy**

Biofilm formation was observed both under a fluorescent microscope (Eclipse LV100D, Nikon Inc. NY) and scanning electron microscope (SEM). For fluorescent microscopy, electrodes were fixed in 4 % paraformaldehyde for 30 min at room temperature, rinsed with PBS (pH 7.4) and stained with 5 μg/ml DAPI (4', 6-diamidino-2-phenylindole) for 5 min, and followed by a final wash in PBS (pH 7.4) for 5 min before microscopy. Microscopic photographs were taken with an Olympus BX51 fluorescent microscope (Olympus America, NY) equipped with an Olympus DP70 camera.

For SEM, electrodes were fixed in sodium phosphate buffer (0.05 M, pH 6.8) with 3 % glutaraldehyde for 90 min, and rinsed thoroughly in 0.05 M sodium phosphate buffer (pH 6.8) for 90 min. Next, electrodes were placed in 2 % osmium tetroxide (in 0.05 M phosphate buffer, pH 6.8) for 2 hr and then dehydrated in ethanol, followed by drying in a critical point drier.

**Assembly of the MFC Array**

First, each cathode well was filled with 170 μl of cathode solution (100 mM ferricyanide in PBS) (Fig. 2*C*) followed by placing the PEM and the anode well layer on top. The acrylic part of the anode well layer was then screwed together with the bottom acrylic frame. Microbe containing solution (650 μl) was pipetted into each anode well (Fig. 2*D*). Finally, the anode electrode layer was flipped over and then the top acrylic frame was screwed with the bottom acrylic frame (Fig. 2*E*).

**Supplementary References**

1. Siripong S & Rittmann BE (2007) Diversity study of nitrifying bacteria in full-scale municipal wastewater treatment plants. *Water Res* 41(5):1110-1120.

2. Thompson JD, Higgins DG, & Gibson TJ (1994) Clustal-w — improving the sensitivity of progressive multiple sequence alignment through sequence weighting, position-specific gap penalties and weight matrix choice. *Nucleic Acids Res* 22(22):4673-4680.

**Supplementary Figures**

**Figure S1. Power density vs. current density from an MFC with carbon cloth anode (n = 3).**

**Figure S2. Microscopy images of Au electrode.** (A) After 1 hour of usage (light microscope). (B) After 5 hours of usage (fluorescent microscopy, DAPI staining). Microbes attached to the gold electrode could be clearly observed. (C) & (D): Scanning electron micrographs of microbes attached to the surface of the gold electrode after 5 hours in an MFC.

**Figure S3.** **Fabrication steps of the MFC array.** (A) Electrode layer (both cathode and anode) fabrication steps. 1. Titanium deposition; 2. Gold deposition; 3. Photoresist (PR) spin coating; 4. UV exposure of PR through a lithography mask; 5. PR developing; 6. Au and Ti etching; 7. PR removing. (B) PDMS layer fabrication steps via softlithography for cathode and anode well layers. 1. Acrylic master mold fabrication using a rapid prototyping tool; 2. PDMS mixing and pouring onto the acrylic master mold; 3. PDMS curing and peeling off.

**Figure S4. Phylogenetic tree based on 16S rDNA sequences showing relationship within of the environmental isolates obtained in the pre-screening. Most environmental isolates were members of classes *Bacilli* or γ-proteobacteria.**

**Figure S5.** **Polarization curves of 7Ca (blue) and *S. oneidensis* MR-1 (SO, red) in the MFC array.**
