# Supplementary figures and images for: Microfabricated Microbial Fuel Cell Arrays Reveal Electrochemically Active Microbes
Source: PLoS One. 2009 Aug 10;4(8):e6570. doi: 10.1371/journal.pone.0006570 (PMC2718701; doi:10.1371/journal.pone.0006570)

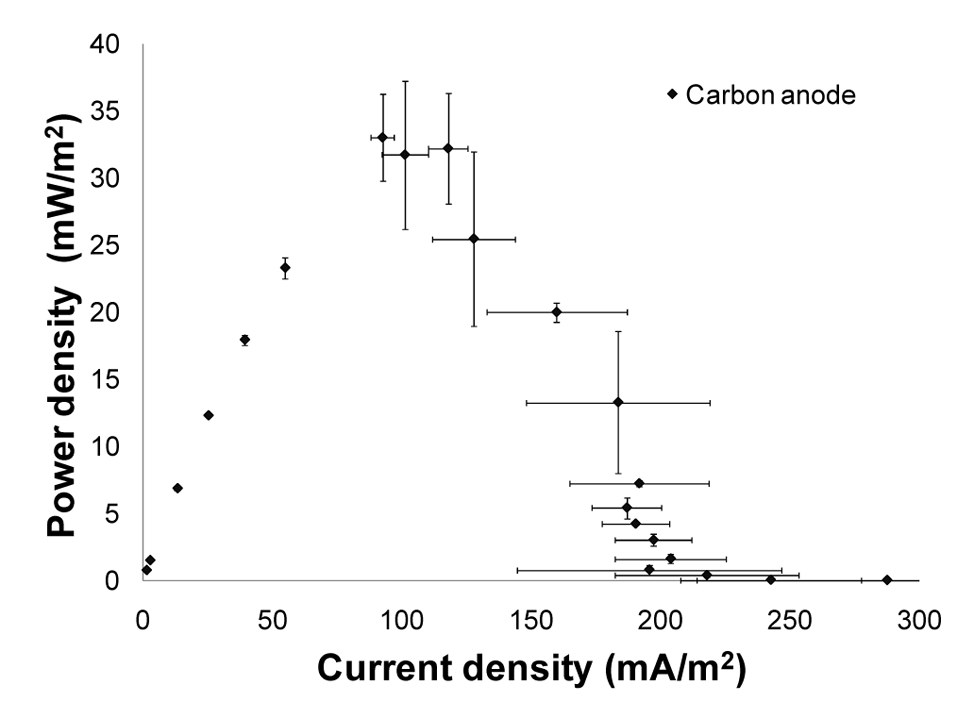

Supplement: Figure S1 — Power density vs. current density from an MFC with carbon cloth anode (n = 3). (0.39 MB TIF) [file pone.0006570.s002.tif]

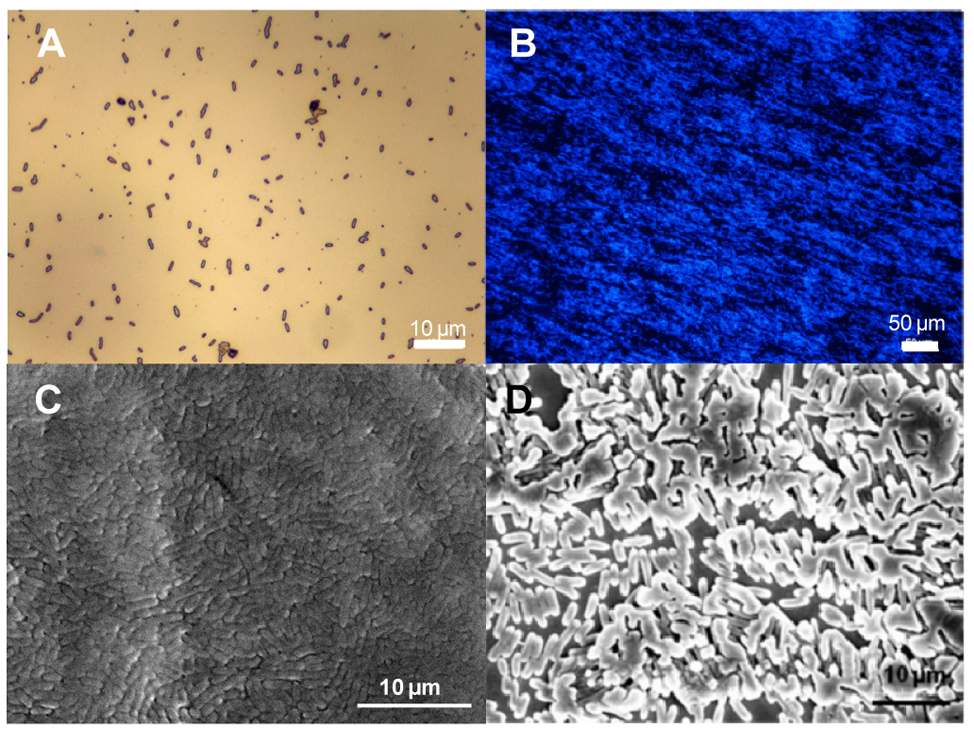

Supplement: Figure S2 — Microscopy images of Au electrode. (A) After 1 hour of usage (light microscope). (B) After 5 hours of usage (fluorescent microscopy, DAPI staining). Microbes attached to the gold electrode could be clearly observed. (C) & (D): Scanning electron micrographs of microbes attached to the surface of the gold electrode after 5 hours in an MFC. (4.63 MB TIF) [file pone.0006570.s003.tif]

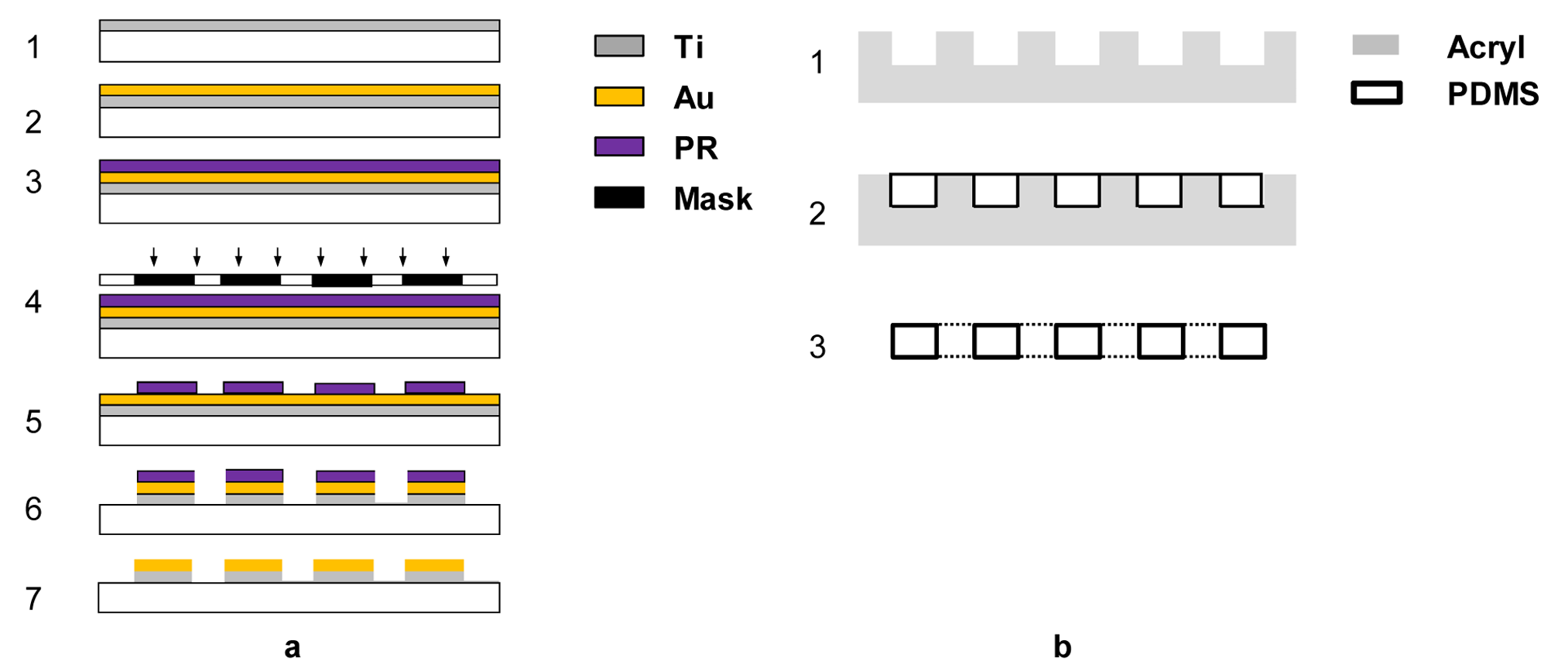

Supplement: Figure S3 — Fabrication steps of the MFC array. (A) Electrode layer (both cathode and anode) fabrication steps. 1. Titanium deposition; 2. Gold deposition; 3. Photoresist (PR) spin coating; 4. UV exposure of PR through a lithography mask; 5. PR developing; 6. Au and Ti etching; 7. PR removing. (B) PDMS layer fabrication steps via softlithography for cathode and anode well layers. 1. Acrylic master mold fabrication using a rapid prototyping tool; 2. PDMS mixing and pouring onto the acrylic master mold; 3. PDMS curing and peeling off. (0.45 MB TIF) [file pone.0006570.s004.tif]

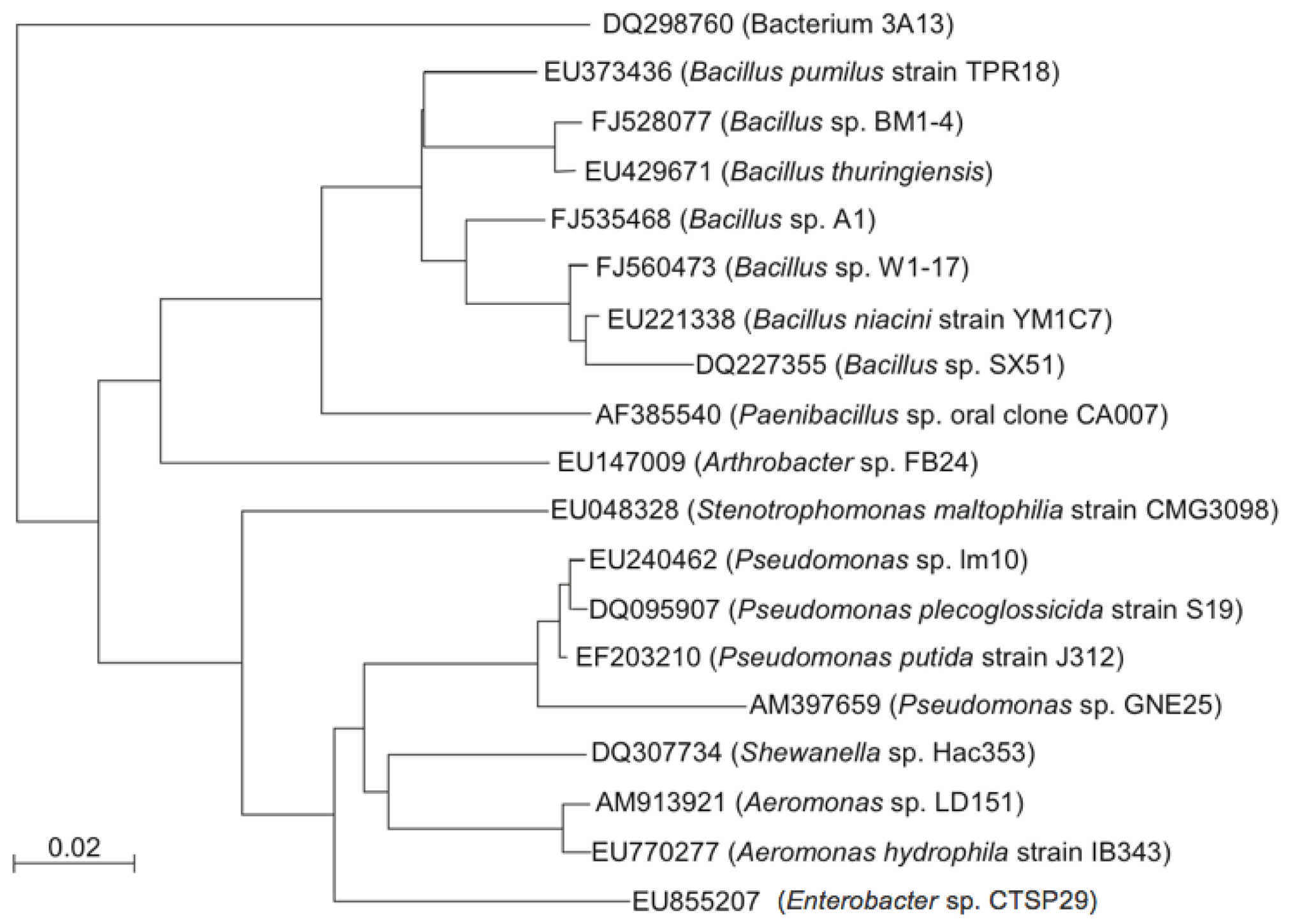

Supplement: Figure S4 — Phylogenetic tree based on 16S rDNA sequences showing relationship within of the environmental isolates obtained in the pre-screening. Most environmental isolates were members of classes Bacilli or γ-proteobacteria. (2.65 MB TIF) [file pone.0006570.s005.tif]

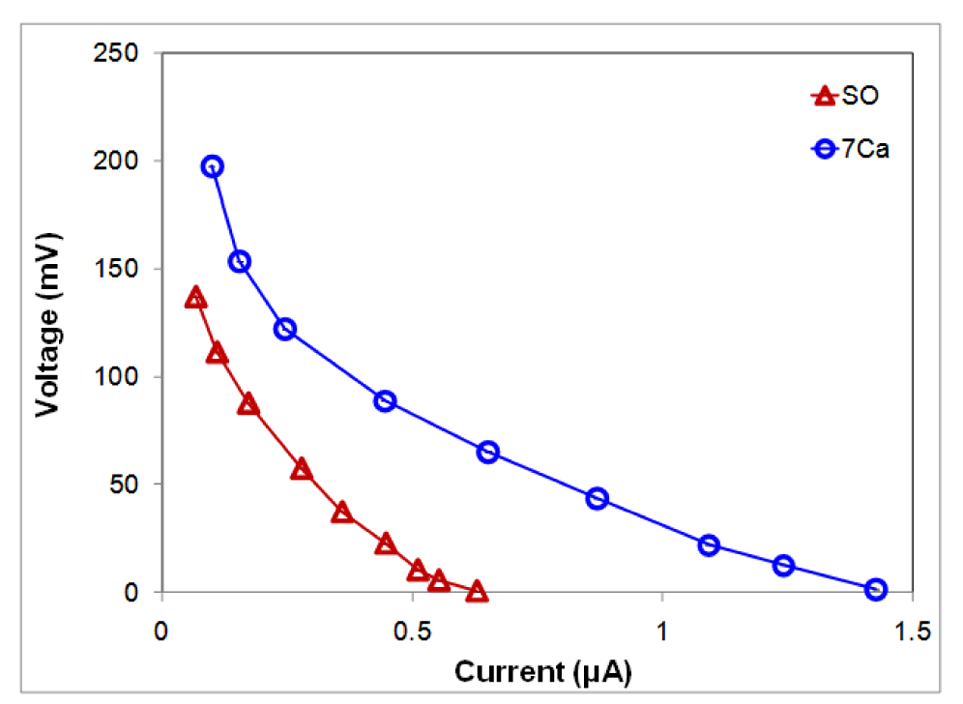

Supplement: Figure S5 — Polarization curves of 7Ca (blue) and S. oneidensis MR-1 (SO, red) in the MFC array. (0.12 MB TIF) [file pone.0006570.s006.tif]
